# Estimated mortality attributable to the urban heat island during the record-breaking 2022 heatwave in London

**DOI:** 10.1088/1748-9326/ad6c65

**Published:** 2024-08-20

**Authors:** Charles H Simpson, Oscar Brousse, Clare Heaviside

**Affiliations:** Institute for Environmental Design and Engineering, University College London, London, United Kingdom

**Keywords:** urban heat island, heatwaves, attributable mortality, air pollution

## Abstract

The United Kingdom experienced its most extreme heatwave to date during late July 2022, with maximum air temperatures exceeding 40 °C recorded for the first time in history on July 19th. High ambient temperatures have been statistically shown to lead to increased mortality. Higher nighttime temperatures that occur in more urbanised areas, called the urban heat island (UHI), may contribute to the mortality burden of heat. In this study, we applied health impact assessment methods with advanced urban climate modelling to estimate what contribution the UHI had on the mortality impact of the 10–25 July 2022 heatwave in Greater London. Estimated mortality due to heat and due to the UHI were compared with estimated mortality due to air pollution in the same period, based on monitored concentrations. We estimate that of the 1773 deaths in Greater London in this period 370 (95% confidence interval 328–410) could be attributed to heat. We estimate that 38% of these heat-related deaths could be attributed to the UHI. In the same period is estimate deaths attributable to PM2.5 were 20.6 (10.4–30.8) and to ozone were 52.3 (95% confidence interval 18.6–85.2). Despite not contributing to the record-breaking maximum air temperature observed during this period, the UHI may have contributed to the heatwave’s mortality burden through raised nighttime temperature. While air pollutant concentrations were elevated during the period, deaths attributable to air pollution were relatively few compared to deaths attributable to heat.

## 1. Introduction

Periods of extreme temperature lead to increased mortality, especially among older adults, and many studies have established statistical relationships between temperature and excess mortality (e.g. (Arbuthnott *et al* 2020, Gasparrini *et al* 2022)). There is increasing awareness of heatwaves as a public health issue (Ebi *et al* 2004, Mitchell and Lo 2022).

Summer 2022 saw several heatwaves across Europe and North Africa driven by high-pressure weather systems. Across Europe, a previous study attributed 62 thousand excess deaths to heat over summer 2022, with the United Kingdom (UK) having one of the highest estimated heat-related mortality counts (Ballester *et al* 2023). There were three periods in which heat-health alerts were issued by the in the United Kingdom (UK) Met Office, in the summer of 2022: 16–19 June, 10–25 July, and 8–17 August (UK Health Security Agency 2024). The most extreme was the second of these, and on July 19th temperatures exceeding 40 °C were recorded for the first time in the UK, exceeding previous records by a large margin (1.6 °C) (Kendon *et al* 2023)—something that could almost certainly be attributed to anthropogenic climate change (Philip *et al* 2020, Zachariah *et al* 2022). Between 10–25 July, the UK recorded 2227 excess deaths (10.4% above average for the period; UK Office for National Statistics and UK Health Security Agency 2022). Between June and August 2022, the day with the greatest number of recorded deaths in Greater London was July 19th (Office for National Statistics 2022).

More urbanised areas tend to have higher ambient air temperatures on average than less urbanised areas (a phenomenon called the urban heat island (UHI) (Oke 1982)). Mechanisms have been proposed by which UHIs and heatwaves could interact through altered windspeed or moisture availability, meaning that UHI intensity will change according to background conditions (Oke 1982, Li and Bou-Zeid 2013). As UHI effects are most prominent at night, they would not be expected to have contributed to the record-breaking maximum air temperature during this period. However, both daytime and nighttime temperatures are thought to contribute to the mortality burden of heat. Analyses of contributions of heat related mortality associated with UHIs in Birmingham and London have estimated up to half of mortality could be attributed to the UHI (Heaviside *et al* 2016, Simpson *et al* 2023) based on the UHI’s effect on the daily-mean temperature.

Previous studies have demonstrated a positive relationship between temperature and air pollution levels (PM2.5 and ozone) and have estimated the mortality impact of the additional air pollution: for example, (Stedman 2004) estimated that 21%–38% of excess deaths in England and Wales during the August 2003 heatwave might be attributable to elevated air pollution. Some had previously suggested that elevated air pollution, rather than the direct effects of temperature, could explain a large proportion of excess deaths during heatwaves (for example, (Rooney *et al* 1998)).

Estimates of the mortality impact of heatwaves (in the past or future) require estimates of exposure, often based on observations from weather stations outside city centres (for the past) or modelled temperatures (for the past or future). Non-urban observations cannot well represent urban conditions, although this is sometimes partly compensated for through land-use regression as in the widely used HadUK-Grid (Hollis *et al* 2019). Many studies have made use of remote observations of surface temperature to characterise the thermal environment of cities, but in reality the relationship between air temperature and surface temperature is complex (Chakraborty *et al* 2022). Use of non-urban observations can mean that health impact assessments underestimate the impact of heatwaves in urban areas (Heaviside *et al* 2016).

Regional climate models however offer opportunities related to their embedded urban canopy models which represent the urban effect on temperature with acceptable performance. In combination with urban crowdsourced weather observations from personally owned weather stations (PWS)—which offer greater capacity to reveal urban microclimatic variations in much greater detail than is possible with official weather observations only, and despite their lower accuracy—(Chapman *et al* 2017, Napoly *et al* 2018, Brousse *et al* 2022, 2023) to identify and correct where models perform less well and address this lack of urban-specific information (Brousse *et al* 2023). As climate projections over the UK indicate that heatwaves would increase in frequency and intensity in future (Lowe *et al* 2018), it is important to estimate how much of the excess deaths observed during heat waves are related to heat and how many were added by the UHI. This study therefore uses advanced urban climate modelling to examine the impact of urban climate effects on exposure to high temperatures in London during the record-breaking heatwaves of July 2022. Official and personal weather station observations are used to evaluate model performance, and comparisons are made to alternative data sources.

In this study we aim to address a research gap around the contribution of the UHI to heat-related mortality during extreme heatwaves, examining London’s first 40 °C heatwave specifically. Estimates of the impact of urban climate effects on mortality are presented and are compared with estimated effects of air pollution on mortality during the same period.

## 2. Method

Urban temperatures were simulated using the *Weather Research and Forecast* (WRF; version 4.3) regional climate model (Chen *et al* 2011), using the *Building Effect Parameterization* model with its coupled *Building Energy Model* (Martilli *et al* 2002) to represent urban areas. For this study the urban heat island intensity (UHII) is defined relative to a modelled counterfactual in which urban areas do not affect the local climate (called the ‘non-urban scenario’), rather than as the difference between urban and rural locations. Each location within the model therefore has an UHII, with the advantage being that it avoids the issue of the local climate of rural reference locations being influenced by nearby urban areas. For the non-urban scenario, the urban land surfaces in the model are changed to with mixed grassland and cropland.

In all respects apart from the period of simulation the present study uses the same modelling setup and validation strategy as described in (Brousse *et al* 2023, section 2a). The key points of the setup are as follows: (i) A two-way nesting strategy was used, with the inner domain at 1 km horizontal resolution centred on London; (ii) the outer domain was forced by ERA5 6-hourly data (Hersbach *et al* 2020), (iii) urban canopy parameters for both the urban and non-urban scenario were generated using the WUDAPT-TO-WRF python tool, based on the European local climate zone mapping generated by the WUDAPT project (Brousse *et al* 2016, Demuzere *et al* 2019, 2022) the Bougeaut-Laccarere planetary boundary layer scheme was used (Bougeault and Lacarrere 1989); and (iv) the model was evaluated against both official stations coming from the MIDAS Open network as well as personal weather stations from the Netatmo weather station network. More information on the urban climate model setup and validation can be found in Brousse *et al* (2023). The model outputs are archived on Zenodo 10.5281/zenodo.11384953 (Simpson *et al* 2024).

Population weighted mean temperature was calculated using 2021 census population counts at output-area level (Office for National Statistics 2021), re-gridded to the grid used by WRF using conservative area weighting. Only the population within the regional boundary of Greater London was included in this analysis.

Exposure-response functions describe the relationship between an exposure (temperature above a threshold in this case) and a response (risk of mortality). Exposure-response functions derived by (Arbuthnott *et al* 2020) were used to estimate the impact of ambient temperature on mortality; these were derived from an ecological time series regression of regional total daily mortality count against 0–2 day lag mean temperature in London from 1996 to 2013. The threshold was 18.9 °C, which was determined based on the inflection point of a spline fit to the temperature-mortality data, and the effect of temperature on relative risk of mortality was assumed to be log-linear above the threshold. The exposure-response function was calculated separately for age groups 0–64, 65–74, and ⩾75 years old and by sex; for the present study we keep the separate age groups but aggregate sex as we did not have access to recorded mortality statistics stratified by both sex and age. Age grouping was determined by the aggregation of the mortality data and exposure-response functions to which we had access. Equations describing this calculation are given in the Supplementary Material.

Mortality attributable to heat was calculated with the urban and non-urban temperature series, and the mortality attributable to the UHI was estimated as the difference between attributable mortality in the scenarios, following (Heaviside *et al* 2016).

(Arbuthnott *et al* 2020), which provided the exposure-response function, performed a sensitivity analysis which did not find a large effect from adjusting for air pollution or relative humidity, and therefore did not adjust for the effect of these factors on the mortality time series. Air pollution and high temperatures are both often higher in atmospherically stable, low-wind conditions; therefore, our mortality estimate may include some deaths that could equally be attributed to air pollution or to the combined exposure of heat and air pollution. We therefore also present exposure and attributable mortality for PM2.5 and ozone to provide a comparison. Urban background concentrations of ozone, PM10 and PM2.5 were taken from Bloomsbury monitoring station via DEFRA (UK Department for Food and Rural Affairs n.d.) (https://uk-air.defra.gov.uk/networks/site-info?site_id=CLL2, last accessed 29 April 2024). Attributable mortality was estimated for ozone and PM2.5 based on (Committee on the Medical Effects of Air Pollutants (COMEAP) 2018) advice for ozone and (Atkinson *et al* 2014) for PM2.5, based on the daily maximum 8 h running mean of exposure as recommended by (Committee on the Medical Effects of Air Pollutants (COMEAP) 2018). Exposure-response coefficients used were 0.34% (95% confidence interval, 0.12%–0.56%) per 10 *μ*g m^−3^ for ozone (Committee on the Medical Effects of Air Pollutants (COMEAP) 2018), and 1.04% (95% confidence interval 0.52%–1.56%) per 10 *μ*g m^−3^ (Atkinson *et al* 2014). Air pollution was assumed to affect each age group and sex equally. Presented confidence intervals for mortality attributed to air pollution are calculated from the confidence intervals of the exposure-response coefficients.

## 3. Results

### 3.1. Modelled air temperature

Examination of the observational records for the St James’s Park weather station, the most central and urban weather station in London, shows that in terms of daily maximum temperature the 19 July 2022 was the hottest day on record, and the 18 July 2022 was the second hottest day on record. In terms of daily minimum temperature, the 18 July 2022 was the fifth hottest night on record. This shows that the study period was exceptionally hot during both the daytime and the nighttime.

**Figure 1.**
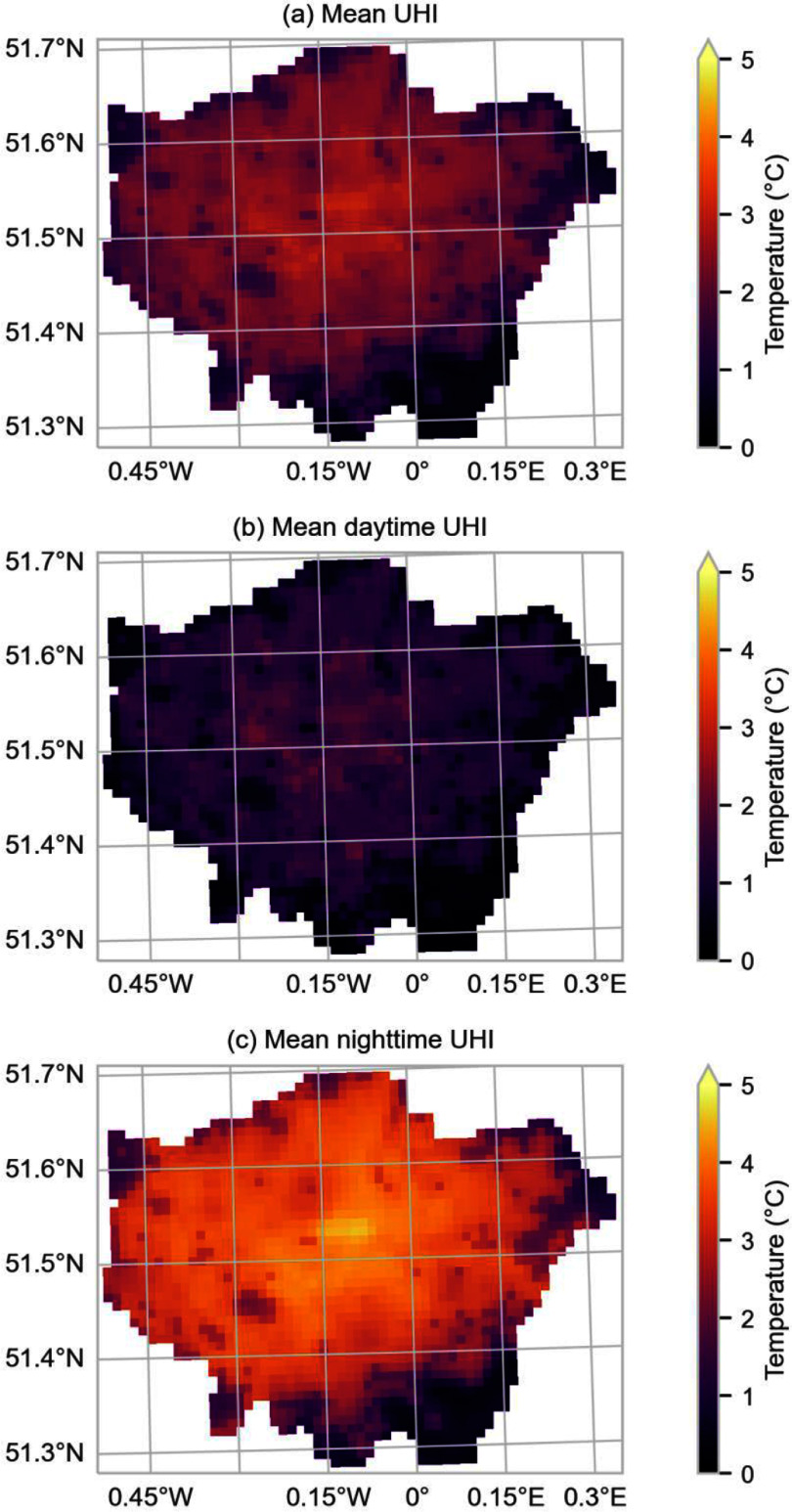
Maps of mean urban heat island intensity, defined as the urban–rural simulation difference. Mean covering the whole simulation period (a), just daytime (8am–8pm) (b) and just nighttime (8pm–8am) (c). Note that the colour scales do not have the same limits.

**Figure 2.**
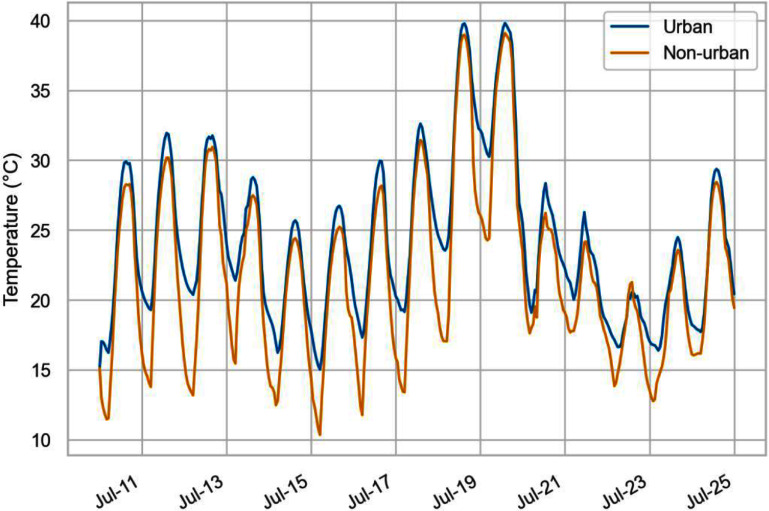
Time series of simulated urban versus non-urban temperatures, hourly mean spatially weighted by population.

Figure 1 shows temperature differences between urban and non-urban simulations within the Greater London region. Figure 2 shows the hourly time series of population-weighted temperature in the urban and non-urban simulations. Population-weighted mean hourly temperatures in the urban simulation had a minimum of 15.0 °C, a maximum of 39.8 °C, and a mean of 24.2 °C in this 15 d period (supplementary table S1). The overall maximum modelled temperature in a single grid cell in Greater London was 41 °C. The difference between the urban and non-urban simulations had a minimum of −1.0 °C (on July 22nd, after the heatwave, the non-urban simulation has a higher maximum temperature than the urban simulation), a mean of 2.3 °C, and a maximum of 7.2 °C. Differences between the urban and non-urban simulations were greatest at night (8pm–8am), and greatest in the centre of London. There were areas in London that were modelled as being warmer due to urban effects during the day.

**Figure 3.**
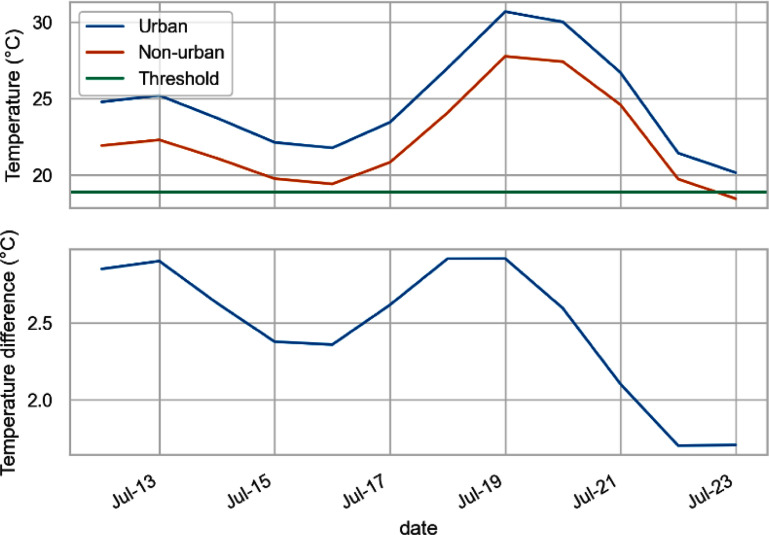
(a) Modelled population-weighted 0–2 d lag mean air-temperature series, with threshold of exposure-response function. (b) Difference in population-weighted 0–2 d lag temperature between urban and rural simulations.

Estimates of heat-related mortality are usually based on daily mean temperature (or lagged daily mean temperature). Figure 3 shows the 0–2 d lag mean temperature, spatially weighted by population. During this period, temperatures in both simulations are above the threshold (19.8 °C) established by (Arbuthnott *et al* 2020). Differences between the urban and non-urban runs are between 2 °C–3 °C before and during the heatwave, declining as the heatwave ends (20th July onward).

When modelled air temperature is compared to observations there is generally a high level of agreement on average, but the spatial distribution of urban temperatures is more uniform in the model than in urban observations. Supplementary table S2 describes the statistics of the comparisons between model and observation. Official MIDAS observations (which tend to be in rural locations on the outskirts of towns and cities) fit with the non-urban simulation, with a mean absolute error of 1.73 °C. Temporal variations have an average Pearson’s R of 0.94 against the non-urban simulation and 0.93 against the urban simulation, indicating that the diurnal cycle of temperature is accurately represented in rural areas in both urban and non-urban simulations. The mean bias of the urban simulation versus urban PWS observations is 0.09 °C. Temporal variations have an average Pearson’s R of 0.88 versus PWS indicating good representation of the diurnal cycle, while the RMSE versus PWS is 3.09 °C. Mean bias is higher for the MIDAS observations against the urban run (0.84 °C) compared to the non-urban run (0.18 °C), showing that improving the urban representation might appear to slightly reduce performance in rural areas and therefore demonstrating the vital role of urban observations. Supplementary figure S2 shows that inclusion of the urban scheme markedly improves the representation of air temperature against urban observations, both spatially and temporally. Supplementary figure S1 suggests that the urban scheme appears to perform slightly worse spatially than temporally, as it seems to underestimate the amount of variation in temperature within urban areas while getting the average temperature correct; this is not a problem for the present study as we regionally average the temperature before making inferences about mortality. Furthermore, we would expect the spread in PWS temperatures to be larger than for standard weather stations as their placement and design is less standardised and calibrated; the urban climate model operates at a 1 km horizontal spatial scale whereas the PWS will be influenced by microclimatic effects at smaller scales. Given the low mean bias and high temporal correlation of both simulations against relevant observations, we determined model performance was sufficient and that bias adjustment as suggested in (Brousse *et al* 2023) would not be necessary.

### 3.2. Estimated mortality

Table 1 presents mortality estimates based on the exposure-response function of (Arbuthnott *et al* 2020). Based on this, we estimate that 370 (95% confidence interval 328–410) deaths were attributable to heat in Greater London between 12th July and 23 July 2022 inclusive, which is 21% of total mortality in this period. We estimate that the difference in heat-related mortality between an urban and non-urban scenario is 141 (95% confidence interval 126–157), which we argue is the portion of mortality attributable to the UHI (8% of total mortality, 38% of heat-related mortality in this period).

**Table 1.** Estimated mortality impact of the heatwave based on modelled temperature and the exposure response function from (Arbuthnott *et al* 2020). Confidence intervals in brackets are 95% and based on the exposure-response function.

	Attributed mortality			
Scenario	Total	Under 65 years	65–74 years	75 years or over
Urban	370	45	39	286
	(328–410)	(32–56)	(29–49)	(266–305)

Non-urban	228	26	23	179
	(202–253)	(19–33)	(17–29)	(166–191)

UHI	141	18	16	107
	(126–157)	(13–23)	(12–20)	(100–114)

Total mortality	1773	371	288	1114

**Figure 4.**
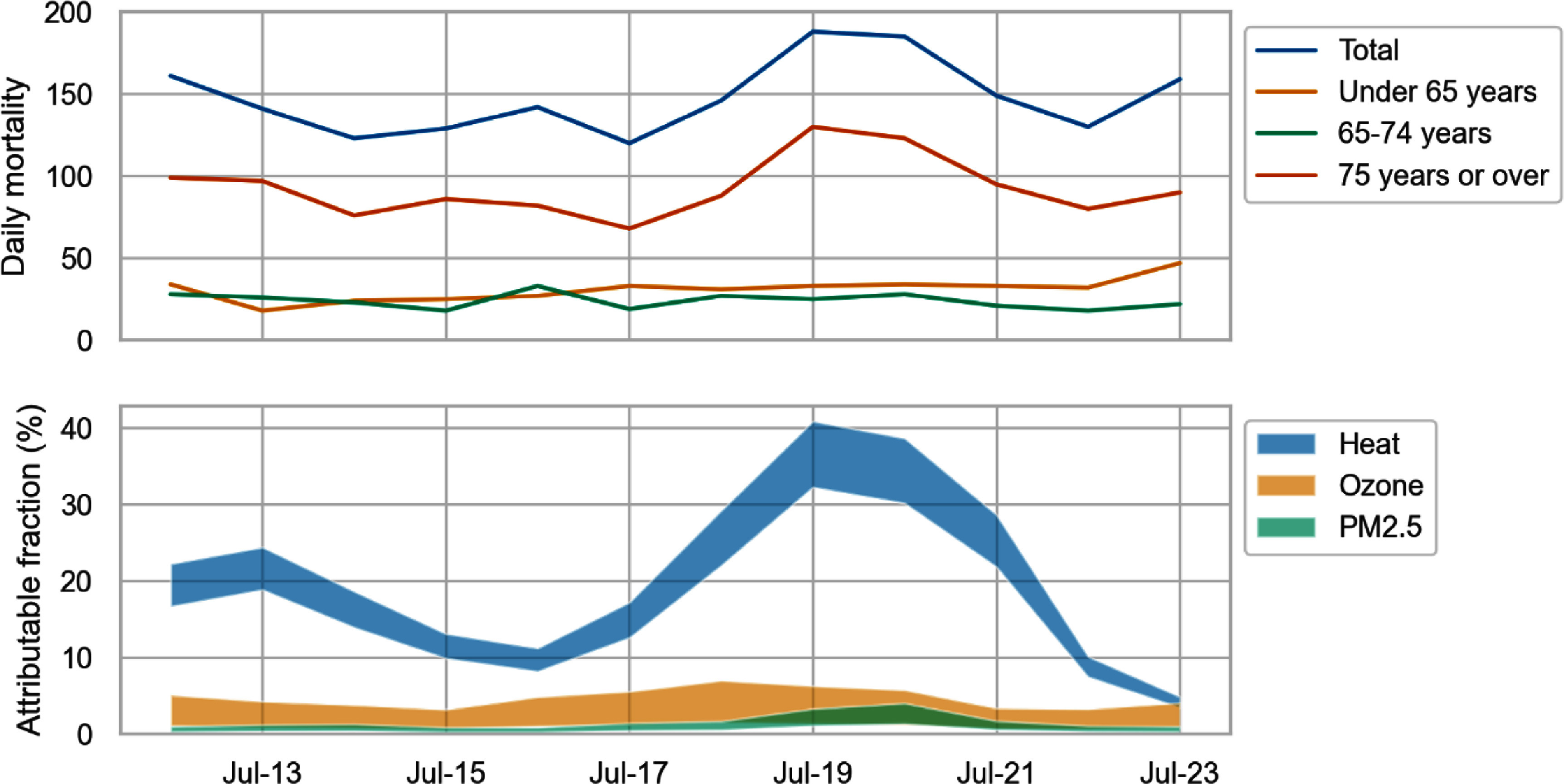
(a) Recorded mortality time series. (b) Comparison of estimated mortality impact of heat, ozone, and PM2.5. Exposure-response coefficients from (Committee on the Medical Effects of Air Pollutants (COMEAP) 2018) for ozone, (Atkinson *et al* 2014) for PM2.5, (Arbuthnott *et al.* 2020) for heat. Exposures based on modelled data (for heat) and Bloomsbury observations for ozone and PM2.5.

Figure 4 shows the time series of the fraction of daily mortality attributable to heat and air pollution exposures, as well as the time series of recorded mortality. Daily exposure numbers to PM2.5 and ozone from Bloomsbury observations are shown in supplementary figure S3. Supplementary figure S4 shows scatter plots of PM2.5 and ozone concentrations against daily maximum temperature for JJA 2024 and JJA 2003. While, for summers in both 2003 and 2022, ozone and PM2.5 concentrations were typically higher on hotter days, PM2.5 concentration was much lower in 2022. On days with a regional average maximum temperature above 30 °C, PM2.5 was a factor of 3 lower in JJA 2022 compared to JJA 2003, while ozone was 17% higher. We estimate 20.6 (95% confidence interval 10.4–30.8) deaths are attributable to PM2.5 (1% of total mortality in the period), and 52.3 (95% confidence interval 18.6–85.2) attributable to ozone (3% of total mortality in the period) between 12th July and 23 July 2022 inclusive. The central estimate of deaths due to heat is 17 times that of PM2.5 and 7 times that of ozone: estimated deaths attributable to air pollution are substantially lower than estimated deaths attributable to heat during this period.

## 4. Discussion

In this study, we estimate that 107 of 286 heat related deaths in Greater London between 12th July and July 23rd in 2022 could be attributed to the UHI. The estimated heat-related mortality count was highest the oldest age group (75 years and older). While the UHI did not contribute to the record-breaking maximum temperature during this period, it may have substantially contributed to heat-related mortality due to elevated daily mean temperatures driven by high nighttime temperatures. Estimated heat attributable mortality (21%) for this period is much higher than estimated air-pollution attributable mortality (1% and 3% for PM2.5 and ozone respectively).

A strength of this study is its use of advanced urban climate modelling in combination with detailed validation against personal weather station data, which provide much greater information about the performance of the model in representing urban areas, which would not usually be available.

We believe this is the first study to explore the effect of urban climate on mortality during the UK July 2022 heatwave. Our central estimate of mortality attributable to heat during the period is slightly above the upper 95% confidence interval of excess mortality estimated by official sources (183 19–349) (UK Health Security Agency 2024) (www.gov.uk/government/publications/heat-mortality-monitoring-reports/heat-mortality-monitoring-report-2022, last accessed 29 April 2024); however, these two estimates use very different methods so should not necessarily be expected to agree.

Our estimates indicate that while PM2.5-related mortality is slightly elevated during and just after the heatwave, many more deaths during this heatwave were attributable to heat rather than air pollution. This contrasts with the August 2003 heatwave during which PM2.5 concentrations were much higher. It is worth noting that an interaction effect on health between ozone concentrations and heat has been hypothesized, which would modify these results (Pattenden *et al* 2010). However, the study from which the exposure-response function used for heat in the present study was taken did not find a significant modifying or confounding effect of air pollution concentration (Arbuthnott *et al* 2020).

A limitation of the approach taken in the present study is that heat-related mortality is assumed to depend only on mean ambient temperature and age: other potential modifiers of heat-related mortality such as air conditioning are not included. Because the 0–2 d lag mean temperature is used to estimate mortality, variations in daytime and nighttime temperatures are assumed to contribute equally to risk of mortality, there is some evidence of differential impacts of daytime and nighttime air temperature which may be relevant as the UHI mainly affects nighttime temperature (Murage *et al* 2017). Some studies have used daily minimum or daily maximum temperatures for estimating the mortality impacts of heat, although there is no statistical indication that any of these provides a consistently better fit to data (Barnett *et al* 2010); choosing to use maximum temperatures would generally reduce the estimated impact of the UHI on mortality. Furthermore, we do not account for any adaptation to higher temperatures that may have occurred between the period of the mortality data (1996–2013) used to estimate the exposure-response function and 2022. There is some evidence that mortality in hotter, more urban areas, while higher than rural areas, is lower than would be expected without acclimatisation, (Milojevic *et al* 2016, Gasparrini *et al* 2022), and there is evidence of populations acclimatising to higher temperatures over time either physiologically or technologically (Arbuthnott *et al* 2016, Achebak *et al* 2019).

A further limitation is that we rely on a single urban climate model to estimate urban effects, and urban observations indicate that this model, while accurately capturing urban–rural differences on average, may produce an unrealistically narrow distribution of intra-urban temperature differences compared to urban PWS observations.

This study raises questions about attribution of events to multiple causes, as this extreme heatwave could not have occurred without certain large scale atmospheric conditions (and global climate change) but equally its impact was exacerbated by urban climate effects at the local scale. The attributed fraction of heat-related mortality to urban climate effects implies that heat-related mortality could be substantially reduced through urban heat mitigation policies, e.g. through urban greening, and that this could continue to be effective in heatwaves with locally unprecedented temperatures. However, completely removing urban climate effects would be extremely ambitious, and acclimatisation could mean that this would have less effect on mortality than expected.

## 5. Conclusion

In summary, we estimate that 107 of 286 heat-related deaths in Greater London between 12th July and 23rd July in 2022 (a record-breaking heatwave period) could be attributed to the UHI, by using urban climate modelling and health impact assessment methods. This is substantially higher than estimated deaths due to air pollution in the same period, despite elevated pollutant concentrations during the heatwave. These results indicate that urban greening could be effective in reducing the health burden of heatwaves, however more research is required into how people acclimatize to long term changes in temperature, and how daytime and nighttime temperatures might affect health differently.

## Data Availability

The data that support the findings of this study are openly available at the following URL/DOI: 10.5281/zenodo.11384953.

## References

[erlad6c65bib1] Achebak H, Devolder D, Ballester J (2019). Trends in temperature-related age-specific and sex-specific mortality from cardiovascular diseases in Spain: a national time-series analysis. Lancet Planet Health.

[erlad6c65bib2] Arbuthnott K, Hajat S, Heaviside C, Vardoulakis S (2016). Changes in population susceptibility to heat and cold over time: assessing adaptation to climate change. Environ. Health.

[erlad6c65bib3] Arbuthnott K, Hajat S, Heaviside C, Vardoulakis S (2020). Years of life lost and mortality due to heat and cold in the three largest English cities. Environ. Int..

[erlad6c65bib4] Atkinson R W, Kang S, Anderson H R, Mills I C, Walton H A (2014). Epidemiological time series studies of PM2.5 and daily mortality and hospital admissions: a systematic review and meta-analysis. Thorax.

[erlad6c65bib5] Ballester J, Quijal-Zamorano M, Méndez Turrubiates R F, Pegenaute F, Herrmann F R, Robine J M, Basagaña X, Tonne C, Antó J M, Achebak H (2023). Heat-related mortality in Europe during the summer of 2022. Nat. Med..

[erlad6c65bib6] Barnett A G, Tong S, Clements A C A (2010). What measure of temperature is the best predictor of mortality?. Environ. Res..

[erlad6c65bib7] Bougeault P, Lacarrere P (1989). Parameterization of orography-induced turbulence in a mesobeta–scale model. Mon. Weather Rev..

[erlad6c65bib8] Brousse O, Martilli A, Foley M, Mills G, Bechtel B (2016). WUDAPT, an efficient land use producing data tool for mesoscale models? Integration of urban LCZ in WRF over Madrid. Urban Clim..

[erlad6c65bib9] Brousse O, Simpson C H, Kenway O, Martilli A, Krayenhoff E S, Zonato A, Heaviside C (2023). Spatially-explicit correction of simulated urban air temperatures using crowd-sourced data. J. Appl. Meteorol. Clim..

[erlad6c65bib10] Brousse O, Simpson C H, Walker N, Fenner D, Meier F, Taylor J, Heaviside C (2022). Evidence of horizontal urban heat advection in London using six years of data from a citizen weather station network. Environ. Res. Lett..

[erlad6c65bib11] Chakraborty T, Venter Z, Qian Y, Lee X (2022). Lower urban humidity moderates heat stress. AGU Adv..

[erlad6c65bib12] Chapman L, Bell C, Bell S (2017). Can the crowdsourcing data paradigm take atmospheric science to a new level? A case study of the urban heat island of London quantified using Netatmo weather stations. Int. J. Climatol..

[erlad6c65bib13] Chen F (2011). The integrated WRF/urban modelling system: development, evaluation, and applications to urban environmental problems. Int. J. Climatol..

[erlad6c65bib14] Committee on the Medical Effects of Air Pollutants (COMEAP) (2018). Mortality Effects of Long-term Exposure to Air Pollution in the UK.

[erlad6c65bib15] Demuzere M, Argüeso D, Zonato A, Kittner J (2022). W2W: a Python package that injects WUDAPT’s Local Climate Zone information in WRF. J. Open Source Softw..

[erlad6c65bib16] Demuzere M, Bechtel B, Middel A, Mills G (2019). Mapping Europe into local climate zones. PLoS One.

[erlad6c65bib17] Ebi K L, Teisberg T J, Kalkstein L S, Robinson L, Weiher R F (2004). Heat watch/warning systems save lives: estimated costs and benefits for philadelphia 1995–98. Bull. Am. Meterol. Soc..

[erlad6c65bib18] Gasparrini A, Masselot P, Scortichini M, Schneider R, Mistry M N, Sera F, Macintyre H L, Phalkey R, Vicedo-Cabrera A M (2022). Small-area assessment of temperature-related mortality risks in England and Wales: a case time series analysis. Lancet Planet Health.

[erlad6c65bib19] Heaviside C, Vardoulakis S, Cai X-M (2016). Attribution of mortality to the urban heat island during heatwaves in the West Midlands, UK. Environ. Health.

[erlad6c65bib20] Hersbach H (2020). The ERA5 global reanalysis. Q. J. R. Meteorol. Soc..

[erlad6c65bib21] Hollis D, McCarthy M, Kendon M, Legg T, Simpson I (2019). HadUK-Grid—a new UK dataset of gridded climate observations. Geosci. Data J..

[erlad6c65bib22] Kendon M, McCarthy M, Jevrejeva S, Matthews A, Williams J, Sparks T, West F (2023). State of the UK climate 2022. Int. J. Climatol..

[erlad6c65bib23] Li D, Bou-Zeid E (2013). Synergistic interactions between urban heat islands and heat waves: the impact in cities is larger than the sum of its parts. J. Appl. Meteorol. Clim..

[erlad6c65bib24] Lowe J A, Bernie D, Bett P, Bricheno L, Brown S, Calvert D, Clark R, Eagle K, Edwards T, Fosser G (2018). UKCP18 Science Overview Report.

[erlad6c65bib25] Martilli A, Clappier A, Rotach M W (2002). An urban surface exchange parameterisation for mesoscale models. Bound. Layer Meteorol..

[erlad6c65bib26] Milojevic A, Armstrong B G, Gasparrini A, Bohnenstengel S I, Barratt B, Wilkinson P (2016). Methods to estimate acclimatization to urban heat island effects on heat- and cold-related mortality. Environ. Health Perspect..

[erlad6c65bib27] Mitchell D M, Lo Y T E (2022). Downplaying the catastrophic health impact of heatwaves costs lives. BMJ.

[erlad6c65bib28] Murage P, Hajat S, Kovats R S (2017). Effect of night-time temperatures on cause and age-specific mortality in London. Environ. Epidemiol..

[erlad6c65bib29] Napoly A, Grassmann T, Meier F, Fenner D (2018). Development and application of a statistically-based quality control for crowdsourced air temperature data. Front. Earth Sci..

[erlad6c65bib30] Office for National Statistics (2021). Census.

[erlad6c65bib31] Office for National Statistics (2022). Daily death occurrences, England and Wales: 2021 and 2022. https://www.ons.gov.uk/peoplepopulationandcommunity/birthsdeathsandmarriages/deaths/adhocs/1724dailydeathoccurrencesenglandandwales2021and2022.

[erlad6c65bib32] Oke T R (1982). The energetic basis of the urban heat island. Q. J. R. Meteorol. Soc..

[erlad6c65bib33] Pattenden S, Armstrong B, Milojevic A, Heal M R, Chalabi Z, Doherty R, Barratt B, Kovats R S, Wilkinson P (2010). Ozone, heat and mortality: acute effects in 15 British conurbations. Occup Environ. Med..

[erlad6c65bib34] Philip S (2020). A protocol for probabilistic extreme event attribution analyses. Adv. Stat. Climatol. Meteorol. Oceanogr..

[erlad6c65bib35] Rooney C, McMichael A J, Kovats R S, Coleman M P (1998). Excess mortality in England and Wales, and in Greater London, during the 1995 heatwave. J. Epidemiol. Community Health.

[erlad6c65bib36] Simpson C H, Brousse O, Heaviside C (2024). Modelled urban climate island during the record-breaking 2022 heatwave in London (Version v01). Zenodo.

[erlad6c65bib37] Simpson C H, Brousse O, Taylor T, Milojevic A, Grellier J, Taylor J, Fleming L E, Davies M, Heaviside C (2023). The mortality and associated economic burden of London’s summer urban heat Island. Preprint.

[erlad6c65bib38] Stedman J R (2004). The predicted number of air pollution related deaths in the UK during the August 2003 heatwave. Atmos. Environ..

[erlad6c65bib39] UK Department for Food and Rural Affairs (n.d.). Site Information for London Bloomsbury (UKA00211).

[erlad6c65bib40] UK Health Security Agency (2024). Heat Mortality Monitoring Report: 2022.

[erlad6c65bib41] UK Office for National Statistics (2022). Excess mortality during heat-periods. https://www.ons.gov.uk/peoplepopulationandcommunity/birthsdeathsandmarriages/deaths/articles/excessmortalityduringheatperiods/englandandwales1juneto31august2022.

[erlad6c65bib42] Zachariah M, Vautard R, Schumacher D L, Vahlberg M, Heinrich D, Raju E, Thalheimer L, Arrighi J, Singh R, Li S (2022). Without human-caused climate change temperatures of 40 C in the UK would have been extremely unlikely. https://researchcommons.waikato.ac.nz/handle/10289/16234.

